# Indocyanine Green Angiography to Predict Complications in Subcutaneous Mastectomy: A Single-Center Experience

**DOI:** 10.3390/jpm15060242

**Published:** 2025-06-10

**Authors:** Letizia Cuniolo, Raquel Diaz, Dafne Anastasia, Federica Murelli, Chiara Cornacchia, Francesca Depaoli, Marco Gipponi, Cecilia Margarino, Chiara Boccardo, Simonetta Franchelli, Marianna Pesce, Amandine Causse D’agraives, Rebecca Allievi, Martina Cossu, Franco De Cian, Piero Fregatti

**Affiliations:** 1 Department of Surgical Sciences and Integrated Diagnostic (DISC), University of Genoa, 16132 Genoa, Italy; 2 Breast Surgery Unit, IRCCS Ospedale Policlinico San Martino, 16132 Genoa, Italy; chiara.cornacchia@hsanmartino.it (C.C.);; 3 School of Medicine, University of Genoa, 16132 Genoa, Italy

**Keywords:** indocyanine green angiography, vascularization, mastectomy, reconstruction

## Abstract

**Background/Objectives**: In the setting of breast surgery, indocyanine green angiography (ICGA) allows estimating the perfusion of cutaneous tissues during surgical interventions, in order to reduce vascularization-related complications. This study has a dual objective: to evaluate the correlation between preoperative factors and the level of skin vascularization, measured by ICGA, in patients undergoing subcutaneous mastectomy for breast cancer; and to establish any relationship between low intraoperative vascularization and the onset of postoperative complications. **Methods**: This is a preliminary, non-randomized, prospective clinical study that includes 46 female patients undergoing subcutaneous mastectomy with reconstruction for breast cancer between February 2022 and July 2024. The relationship between vascularization and the following preoperative variables was assessed: smoking, previous breast surgeries, prior radiotherapy, neoadjuvant or prior chemotherapy/anti-Her2 therapy, and the thickness of breast subcutaneous tissue evaluated through mammography. For the analysis, three ICGA procedures were performed, using 0.125 mg/kg of indocyanine green (ICG) for each procedure before the surgical incision (V1), at the end of the demolition phase (V2), and at the end of the reconstruction phase (V3). The results of this analysis were finally correlated with the occurrence of any postoperative complications. **Results**: Vascularization was conventionally classified as “low” and “good” using a cutoff of 33%. Previous surgeries on the ipsilateral breast and neoadjuvant or prior chemotherapy/anti-Her2 therapy were found to be predictive factors of “low” vascularization (*p* = 0.031). Patients with “low” vascularization at time V3 showed a significantly higher risk of developing complications (*p* = 0.038). Incision length emerged as an independent predictor of complications, with a 23% increase in risk per additional centimeter (*p* = 0.006), independent of perfusion level. **Conclusions**: This study supports the use of ICGA as a useful tool to improve outcomes in patients undergoing subcutaneous mastectomy with prosthetic reconstruction for breast cancer. The results of this preliminary work are encouraging, and recruiting a larger number of patients could provide more significant data.

## 1. Introduction

In recent years, the increasing demand for immediate breast reconstruction after mastectomy has led to significant advancements in surgical techniques and intraoperative assessment tools. Nipple-sparing and skin-sparing mastectomies, combined with prepectoral or retropectoral implant-based reconstruction, have become widely adopted due to their oncological safety and favorable aesthetic results. However, these techniques also pose a risk of postoperative complications, particularly when the vascular supply of the mastectomy skin flaps is compromised. In this context, the evaluation of skin perfusion plays a critical role in minimizing adverse outcomes and improving surgical decision-making.

Traditionally, surgeons have relied on clinical judgment—such as skin color, capillary refill, and dermal bleeding—to assess tissue viability intraoperatively. Nonetheless, these methods are subjective and may fail to detect early signs of hypoperfusion. The introduction of imaging technologies, such as indocyanine green angiography (ICGA), has provided a more objective and reproducible tool for assessing perfusion in real time. By quantifying tissue vascularization, ICGA may help to guide intraoperative choices, such as implant selection, flap revision, or conversion to a staged reconstruction, thereby aligning with the principles of personalized and precision surgery.

In oncological breast surgery, the assessment of skin and subcutaneous tissue perfusion is essential for predicting postoperative complications and improving reconstructive outcomes. The emerging field of personalized medicine emphasizes tailoring medical care to individual patient characteristics, including genetic, environmental, and treatment-related factors. By integrating patient-specific variables into surgical planning, personalized medicine aims to enhance outcomes and minimize risks, providing a patient-centered approach that goes beyond standardized care protocols. In this context, ICGA has proven to be a valuable intraoperative tool. ICGA offers real-time, objective visualization of tissue vascularization, allowing surgeons to assess perfusion dynamically and adapt surgical strategies accordingly, thus contributing to precision medicine in breast cancer surgery [1,2].

ICGA involves the intravenous administration of indocyanine green (ICG) and near-infrared light stimulation. Upon injection, ICG binds rapidly to plasma proteins, fluorescing under near-infrared illumination. The dye has a short plasma half-life (3–4 min) and is excreted via the biliary system without further metabolism. This technology enables real-time evaluation of perfusion across different surgical stages, helping to customize the surgical approach based on the individual patient’s tissue characteristics and needs [3]. Moreover, its ability to provide quantifiable perfusion data facilitates a more nuanced understanding of patient-specific vascular dynamics, aligning with the principles of tailored medical interventions.

This study investigates the role of ICGA in subcutaneous mastectomy with prosthetic reconstruction for breast cancer, focusing on two main objectives that align with the principles of personalized medicine. The primary endpoint is to evaluate whether specific preoperative variables—such as prior surgery, radiotherapy, chemotherapy, subcutaneous thickness on mammography, diabetes, and smoking—correlate with intraoperative vascularization levels. These variables not only reflect the heterogeneity of patient profiles but also highlight the importance of stratifying risks based on individual characteristics. These factors, which vary across individuals, can influence tissue perfusion and, subsequently, the risk of complications. The secondary endpoint is to determine whether low perfusion values, as detected by ICGA, are associated with a higher incidence of postoperative complications, such as infection, skin necrosis, wound dehiscence, or reoperation. This objective underscores the potential of ICGA as a tool to predict complications based on individualized perfusion thresholds. In doing so, it exemplifies the broader goal of personalized medicine: transforming clinical data into actionable strategies that enhance patient outcomes.

## 2. Materials and Methods

This prospective, non-randomized observational study included female patients who underwent subcutaneous mastectomy with immediate prosthetic reconstruction for breast cancer at the Breast Surgery Unit of the San Martino Polyclinic Hospital in Genoa, between February 2022 and July 2024.

Inclusion criteria were women aged 25–76 years undergoing nipple-sparing, skin-sparing, or skin-reducing mastectomy for breast cancer with immediate prosthetic reconstruction. Exclusion criteria included known allergy to ICG, sodium iodide, or iodine, and mastectomies not followed by prosthetic reconstruction.

All procedures were conducted under general anesthesia using standard protocols. Perioperative antibiotic prophylaxis with a single dose of cefazolin 2 g was administered intravenously 30 min before incision, in accordance with institutional guidelines. In patients allergic to beta-lactams, clindamycin was used as an alternative. Thromboprophylaxis with low-molecular-weight heparin was administered postoperatively according to risk stratification.

**Figure 1:**
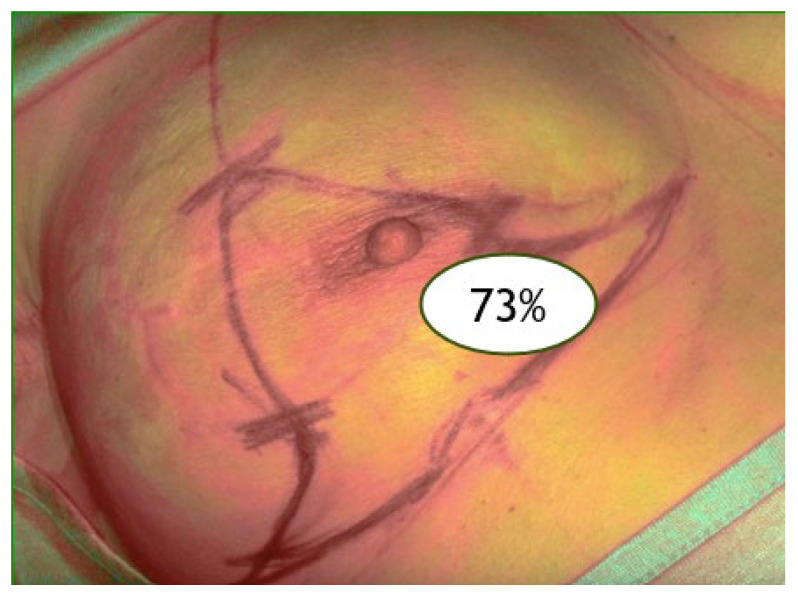
ICGA before the surgical incision (V1).

**Figure 2:**
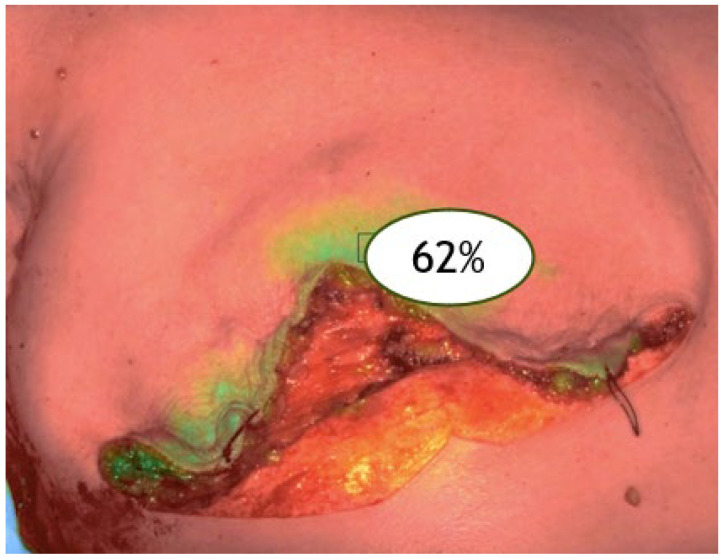
ICGA at the end of the surgical procedure (V2).

**Figure 3:**
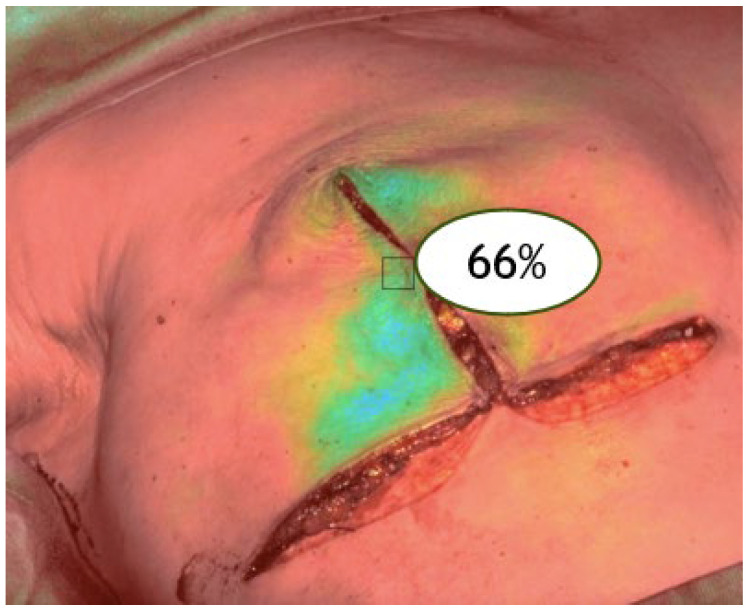
ICGA at the end of the reconstruction (V3).

For each patient, ICGA was performed at three surgical timepoints:

- ICGA time 1: before skin incision (baseline skin vascularization, V1) (Figure 1).

- V2: after mastectomy (post-excision perfusion) (Figure 2).

- V3: after reconstruction (final perfusion post-implant) (Figure 3).

ICG (25 mg vials) was reconstituted in sterile water and administered at 0.125 mg/kg per injection. The SPY-PHI system (Stryker©, 1941 Stryker Way, Portage, MI 49002, USA) was used to obtain fluorescence-based perfusion images, enabling real-time quantitative assessment of tissue vascularization. Images were acquired under low ambient light to minimize interference, with the SPY-PHI device held at a fixed distance of 20–25 cm from the surgical field. Perfusion values were calculated using the system’s built-in software (version 3.0), which expresses relative fluorescence intensity as a percentage compared to the brightest area in the field of view. The time from injection to visualization was approximately 30–60 s, depending on individual patient factors.

All ICGA procedures were performed by trained surgeons familiar with the imaging protocol, ensuring consistency in image acquisition and interpretation. No adverse reactions to ICG administration were recorded in the study population.

Preoperative variables included age, weight, comorbidities, ASA score, smoking status, subcutaneous thickness, prior ipsilateral surgery, previous or neoadjuvant therapies, tumor biology, and BRCA mutation status. These variables were selected to encompass a broad spectrum of factors influencing tissue health and perfusion, aligning with the personalized medicine framework that seeks to integrate genetic, environmental, and clinical data into patient care. Subcutaneous tissue thickness was assessed on preoperative mammography by measuring the distance between the skin and the anterior surface of the gland at the upper outer quadrant, as this area provides a reproducible landmark.

Intraoperative data included type of mastectomy, incision length, implant characteristics, use of dermal matrices, and number of drains. This detailed intraoperative dataset highlights the complexity of surgical planning and the importance of tailoring techniques to individual patient needs. Implant selection was based on preoperative planning considering breast volume, skin quality, and oncological factors. Expanders were used in cases with higher risk of tension or compromised perfusion, while definitive implants were reserved for low-risk cases with favorable intraoperative findings. Dermal matrices (human or bovine) were used selectively to reinforce lower pole coverage, particularly in prepectoral reconstructions.

Postoperative data encompassed complications, number of dressings, adjuvant therapies, and histopathological findings. All patients were monitored daily during hospitalization, and postoperative complications were recorded prospectively up to 12 months.

For the statistical analysis, categorical variables were analyzed using the chi-square test or Fisher’s exact test, as appropriate. Cox regression and Kaplan–Meier analysis were employed to evaluate predictors of complications and time-to-event outcomes. A *p*-value < 0.05 was considered statistically significant.

## 3. Results

A total of 46 patients were included in the study. Regarding preoperative variables, the mean age was 51 years, and the mean BMI was 22.4. Among the patients, 13 had an ASA score of 1, 31 an ASA score of 2, and 2 an ASA score of 3, while none presented with an ASA score of 4 or 5. Fifteen patients were smokers. The mean subcutaneous thickness measured on preoperative mammography was 9 mm.

Eight patients had previously undergone breast-conserving surgery followed by radiotherapy for ipsilateral breast cancer. Of these, three patients had a history of hormonal therapy, three chemotherapy, one chemotherapy combined with anti-Her2 therapy, and one chemotherapy combined with anti-Her2 therapy and hormonal therapy. Genetic testing revealed BRCA1 mutations in two patients and BRCA2 mutations in another two patients.

Concerning tumor histology, 30 patients had Invasive Ductal Carcinoma (IDC), 8 had Invasive Lobular Carcinoma (ILC), and 8 had Ductal Carcinoma In Situ (DCIS). Among those with invasive histotypes, 14 had Luminal A tumors, 10 had Luminal B tumors, 4 had Luminal Her2-positive tumors, 4 had non-luminal Her2-positive tumors, and 6 had triple-negative carcinoma.

Regarding surgical interventions, 19 patients underwent nipple-sparing mastectomy (NSM) with retropectoral expander reconstruction (2 of whom also required decellularized human dermal matrix use), 3 had skin-sparing mastectomy (SSM) with retropectoral expander reconstruction, 5 had NSM with prepectoral implant reconstruction using bovine pericardial matrix, 11 underwent NSM with retropectoral implant reconstruction combined with decellularized human dermal matrix, 3 underwent skin-reducing nipple-sparing mastectomy (SR NSM) with retropectoral Becker implant reconstruction, and 4 patients with BRCA mutations had bilateral NSM (risk-reducing contralateral mastectomy) with retropectoral implant reconstruction and dermal matrix use. The average total duration of the surgical procedure was 136 min, while the mastectomy and reconstruction lasted an average of 48 and 56 min, respectively. The mean volume of the definitive prostheses used was 284.7 milliliters (mL), and the mean intraoperative fill volume of the expanders was 126.5 mL. The mean number of drains used was 2.

Surgical incisions included lazy S in 20 patients, inverted T in 9, and inframammary fold in 17 patients, with a mean incision length of 13.4 cm.

The mean number of dressings required postoperatively was 6.

Complications comprised the following:

- Seroma formation in seven patients: two following NSM with retropectoral expander reconstruction and human dermal matrix; one after SSM with retropectoral expander reconstruction; one after NSM with prepectoral implant reconstruction using bovine pericardial matrix; two after NSM with retropectoral implant reconstruction combined with decellularized human dermal matrix; and one following SR NSM with retropectoral Becker implant reconstruction.
- Superficial skin distress, not requiring reoperation, in three patients: one after NSM with retropectoral expander reconstruction; one after SSM with retropectoral expander reconstruction; and one after NSM with retropectoral implant reconstruction combined with decellularized human dermal matrix.
- Mastectomy flap necrosis in two patients, both necessitating implant removal: one after NSM with prepectoral implant reconstruction using bovine pericardial matrix and one after NSM with retropectoral implant reconstruction combined with decellularized human dermal matrix.

**Figure 4:**
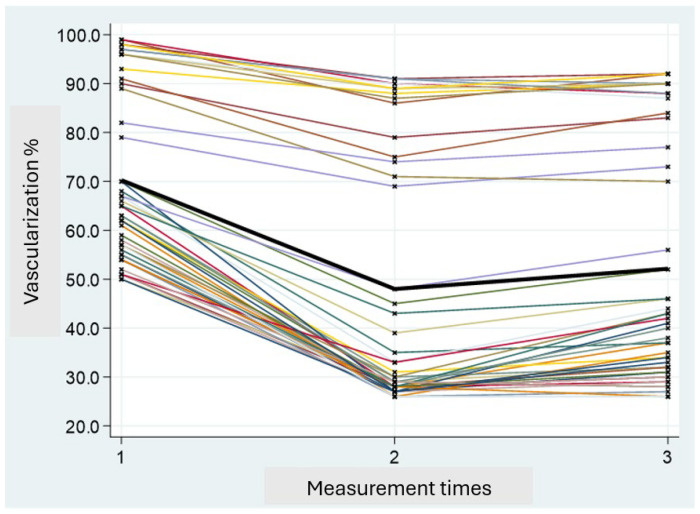
Trend of vascularization values in the three measurements (V1, V2, and V3). The colored lines connect the values for each individual subject, while the thicker black line represents the average trend.

Perfusion values were classified as “low” (<33%) or “good” (≥33%) based on intraoperative ICGA assessments. The percentage of vascularization at V1 in all subjects was higher compared to that observed at times V2 and V3. Specifically, a peak reduction in vascularization was noted at time V2, followed by a partial recovery at time V3. Since there is a correlation between vascularization at V1 and V3, the statistical analysis was conducted based on the V3 values (Figure 4).

Regarding the first endpoint, linear regression analysis revealed that previous ipsilateral surgery combined with radiotherapy and chemotherapy/anti-Her2 therapy, as well as neoadjuvant systemic therapy (chemotherapy/anti-Her2 therapy), were significantly associated with reduced vascularization at V3. Patients with these risk factors exhibited a mean 21.86% and 21.43% decrease in V3 perfusion, respectively (*p* = 0.031 and *p* = 0.002) (Table 1).

Concerning the secondary endpoint, the analysis obtained with the Log-rank test shows the probability of having a complication at 1, 6, and 12 months after surgery in the groups of patients with “low” and “good” vascularization. The difference between the two groups was found to be statistically significant (*p* = 0.038). For example, the value 0.21 (1–0.79) indicates that subjects with vascularization less than 33% have a 21% probability of having complications at one month, compared to 3% in subjects with vascularization equal to or greater than 33% (Table 2).

**Figure 5:**
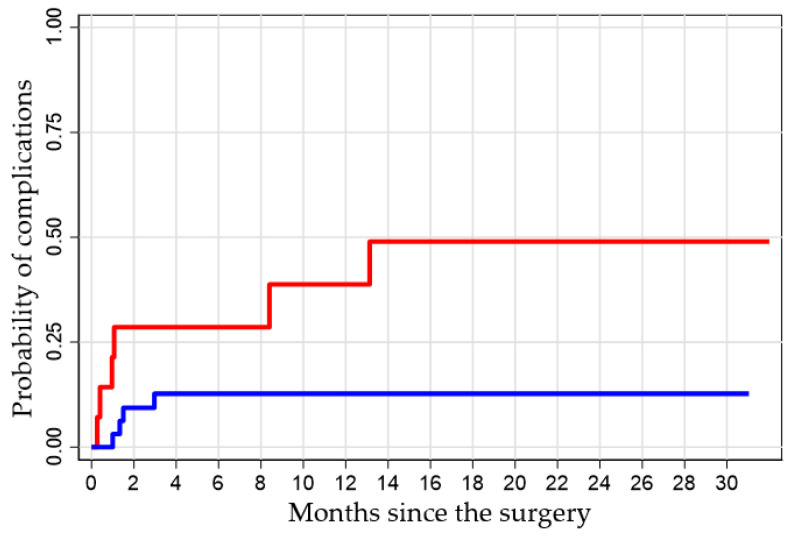
Kaplan–Meier analysis related to vascularization at time V3 and the development of complications: red line: low vascularization; blue line: good vascularization.

Kaplan–Meier analysis demonstrated a significantly higher cumulative incidence of complications in the low-perfusion group (*p* = 0.038). At one month post-surgery, patients with low V3 perfusion had a 21% probability of developing complications, compared to only 3% in the good-perfusion group. By 14 months, complication rates reached 50% versus 13%, respectively (Figure 5).

A Cox regression model identified incision length as an independent predictor of complications: for each additional centimeter of surgical incision, the risk of complications increased by 23% (*p* = 0.006), regardless of perfusion level (Table 3).

## 4. Discussion

Our study confirms the predictive value of indocyanine green angiography (ICGA) in subcutaneous mastectomy with prosthetic reconstruction, highlighting a strong correlation between intraoperative tissue perfusion and the onset of postoperative complications. This reinforces the role of ICGA as a cornerstone in implementing personalized medicine in oncological breast surgery. This observation aligns with the existing literature, where ICGA has proven to be a highly sensitive tool for the early identification of tissue distress areas, with superior sensitivity compared to traditional clinical evaluation [1,2,3,4,5]. Integrating ICGA into surgical practices allows for the personalization of the intraoperative approach, as it enables decisions to be tailored to the specific vascular condition of each patient—an essential aspect of personalized medicine.

The sequential analysis of the three surgical phases revealed a predictable pattern: a significant reduction in perfusion after glandular removal (V2), followed by partial recovery at the completion of reconstruction (V3). However, in cases where residual perfusion at the end of the procedure remained low (<33%), a statistically significant increase in the risk of complications was observed, such as skin necrosis, wound dehiscence, and the need for reoperation. These findings provide a quantitative framework for identifying at-risk patients and tailoring interventions to mitigate complications. The perfusion threshold adopted in this study to classify vascularization as “low” (<33%) or “good” (≥33%) is supported by evidence in the current literature. Moyer et al. demonstrated that tissue areas with a perfusion rate ≤25% were nonviable in 90% of cases, while areas with ≥45% perfusion survived in 98% of cases, suggesting an intermediate threshold of 33% as a practical reference point for clinical decision-making [6]. Nevertheless, it is important to recognize that a “grey zone” exists between 25% and 45% relative perfusion, where tissue viability may be unpredictable [6]. Therefore, intraoperative decision-making should not rely solely on quantitative perfusion thresholds but must also take into account the clinical context, individual tissue characteristics, and other surgical findings [7,8].

The reduction in tissue perfusion after preoperative treatments such as prior surgery, radiotherapy, and chemotherapy/anti-Her2 therapy, as well as neoadjuvant therapy, was a significant factor associated with reduced perfusion at V3. It is well known that radiotherapy and chemotherapy negatively impact microcirculation, impairing neoangiogenesis, endothelial function, and tissue regenerative capacity [9,10,11,12,13,14]. The cumulative effect of these treatments, when combined with previous surgeries, appears to have a compounded impact on skin vascularization, making these patients particularly vulnerable to reconstructive complications [15,16]. In this context, ICGA becomes crucial, as it allows for intraoperative risk stratification based on the patient’s vascular condition, enabling the adoption of personalized surgical approaches that reduce the risk of postoperative complications [17,18,19,20,21].

These data suggest the importance of individualized surgical planning, considering not only the oncological characteristics of the tumor but also the patient’s vascular conditions and tissue quality. Personalized surgical treatment based on tissue perfusion could involve, for example, using a reduced-volume prosthesis or selectively employing vascularized flaps or dermal matrices to ensure better prosthetic coverage without compromising tissue viability [22]. Such adjustments, grounded in real-time perfusion assessments, exemplify the integration of precision medicine into surgical practice. Moreover, the ability to monitor perfusion in real time enables the identification of at-risk areas that could benefit from local preventive measures, such as reinforcing edges with tension-relieving sutures, using targeted drains, or applying pro-angiogenic agents topically [23,24,25].

Our study also highlights another significant variable: incision length. Incision length was identified as an independent risk factor for complications, increasing the risk by 23% for every additional centimeter of incision (*p* = 0.006). This finding reflects the impact that surgical technique can have on tissue perfusion, suggesting that longer incisions may create greater tension on the skin flaps and compromise peripheral vascularization, especially in the absence of adequate dermal collateral vessels [26,27]. This underscores the importance of balancing oncological requirements with reconstructive considerations, a hallmark of personalized surgical strategies. However, incision length may also reflect more complex cases that require greater tissue manipulation, increased prosthetic exposure, or the use of biological matrices that, while contributing to prosthetic coverage, could affect pressure distribution and wound biomechanics [28,29]. As a result, personalizing incision length based on tissue quality and vascular conditions could be a useful strategy to minimize risks. It is important to note that the variable “incision length” may also reflect the extent and pattern of skin flap creation, particularly in cases of inverted T incisions that used NS SRM. These incisions inherently involve wider tissue dissection and redistribution of vascular territories, potentially compromising flap viability. Although we did not perform a specific analysis correlating incision pattern or flap design with perfusion values, we acknowledge that this is a relevant factor, and we consider it a valuable direction for future research.

These findings contribute to a growing body of evidence in favor of integrating personalized medicine into reconstructive breast surgery. The systematic use of ICGA in the operating room enables real-time personalization of the surgical approach, improving the quality of outcomes and reducing complication rates. This approach highlights the shift from standardized surgical techniques to precision-based strategies that consider the unique characteristics of each patient, advancing both clinical efficacy and patient-centered care. Personalized medicine, in this case, extends beyond the selection of tailored pharmacological therapies to also include surgical aspects, allowing for the adjustment of every phase of the surgery based on the patient’s unique characteristics.

This study has several limitations that should be acknowledged. Firstly, it is a monocentric study conducted at a single institution, which may limit the generalizability of the results to different clinical settings. Secondly, the sample size was relatively small, potentially reducing the statistical power of the analysis. Thirdly, the observational, non-randomized design exposes the study to possible selection biases and confounding factors. Additionally, the perfusion thresholds used to classify vascularization levels were based on intraoperative assessments rather than universally validated standards, which could affect reproducibility across centers. In addition, long-term outcomes such as implant loss rates and patient-reported aesthetic satisfaction were not systematically evaluated and should be addressed in future studies. Beyond these limitations, it is important to highlight that no substantial intraoperative changes were made based solely on ICGA results. Patients with low perfusion were already preoperatively selected for reconstruction using tissue expanders with a conservative initial fill volume, taking into account risk factors such as previous radiotherapy and comorbidities. Therefore, ICGA served primarily as a supportive diagnostic tool, while final intraoperative decisions were based on comprehensive clinical evaluation and surgeon discretion.

In the two cases of implant loss, both involving permanent implants (one retropectoral and one prepectoral), the intraoperative perfusion values did not indicate the need to alter the reconstructive approach, such as switching to a lower-volume tissue expander. These unexpected postoperative complications suggest that factors beyond ICGA detection may influence outcomes, warranting further investigation in larger cohorts. A detailed postoperative review was conducted, but no substantial modifications to the reconstructive protocol have yet been implemented.

For patients exhibiting perfusion values below the 33% threshold—conventionally defined as “low perfusion”—reconstruction was never deferred. Instead, a tissue expander with low intraoperative fill volume was consistently placed to mitigate complication risks.

Currently, no formal institutional algorithms or standardized guidelines exist at our center to integrate ICGA data into intraoperative reconstructive decisions; thus, choices remain at the surgeon’s discretion. We believe this study provides a solid foundation for the future development of such protocols and algorithms, which may improve standardization, safety, and efficacy in prosthetic breast reconstruction.

Although autologous reconstruction cases with pedicled or free flaps were not included in our study, we recognize the importance of this field. Future research should focus on evaluating ICGA’s role in assessing flap edge perfusion and vascular pedicle adequacy, which could enhance intraoperative decision-making and reduce complications in more complex reconstructive procedures.

Despite these limitations, this study provides a robust foundation for future multicentric, randomized trials that could validate these findings and further integrate personalized medicine into reconstructive breast surgery.

## 5. Conclusions

Indocyanine green angiography proves to be an effective, safe, and easy-to-apply technique for intraoperative skin perfusion evaluation during subcutaneous mastectomy with prosthetic reconstruction. By providing real-time, dynamic data on tissue vascularization, ICGA enhances the ability to tailor surgical strategies based on each patient’s unique vascular profile, a key aspect of personalized medicine. Our monocentric experience shows that low perfusion values at the end of the procedure are significantly associated with an increased risk of postoperative complications, making ICGA a valuable tool for guiding reconstructive decisions and improving surgical outcomes.

The identification of high-risk patients in the operating room allows for the adoption of personalized preventive strategies, such as modulating the type of implant, revising skin flaps, or postponing definitive reconstruction. These strategies, informed by ICGA-derived perfusion data, reflect the growing trend towards precision surgery that considers the unique characteristics of each patient’s anatomy and treatment history. Moreover, the association between preoperative treatments and reduced perfusion highlights the importance of a comprehensive evaluation of the oncological patient, from a personalized medicine perspective that integrates oncology, surgery, radiotherapy, and individual factors.

In light of these results, we believe that the routine integration of ICGA into reconstructive mastectomy protocols could be a valuable ally for future breast surgery, contributing not only to reducing complication rates but also improving aesthetic satisfaction and patients’ quality of life. Further prospective and multicentric studies will be essential to confirm these findings, define optimal perfusion thresholds, and develop decision algorithms based on intraoperative angiography.

## Figures and Tables

**Table 1:** Correlation between preoperative factors and vascularization.

	Coefficient	95% CI	*p*-Value
Previous surgery + radiotherapy + chemotherapy/anti-Her2 therapy	−21.86	−41.56, −2.16	0.031
Neoadjuvant chemotherapy/anti-Her2 therapy	−21.43	−34.30, −8.57	0.002

**Table 2:** Probability of developing a complication at 1, 6, and 12 months. N: number of patients; C/C%: number/percentage of patients who experienced complications during the follow-up period; p50: median follow-up time (months); IQR: interquartile range of follow-up time (months); Prob.: probability of failure; 95% CI: 95% confidence interval for Prob.; *p*-value: probability level associated with the log-rank test.

Vascularization V3	N	C	C%	Follow-Up Median	Follow-Up IQR	1 Month Prob.	1 Month 95% CI	Failure 6 Months Prob.	Failure 6 Months 95% CI	12 Months Prob.	12 Months 95% CI	*p*-Value
Low (v < 33)	14	6	42.8	7.0	1.1–27.4	0.21	0.07–0.53	0.29	0.12–0.59	0.39	0.18–0.71	0.038
Good (v ≥ 33)	32	4	12.5	5.5	3.3–13.4	0.03	0.00–0.20	0.06	0.05–0.30	0.06	0.05–0.30	
Total	46	10	21.7	5.5	3.0–20.1	0.08	0.03–0.22	0.18	0.09–0.32	0.23	0.12–0.42	

**Table 3:** Cox regression model related to vascularization at V3 and incision length.

	Hazard Ratio	95% CI	*p*-Value
Incision length	1.23	1.06–1.43	0.006

## Data Availability

The data presented in this study are available in this article.
